# Eradication of introduced fish allows successful recovery of a stream-dwelling amphibian

**DOI:** 10.1371/journal.pone.0216204

**Published:** 2019-04-30

**Authors:** Jaime Bosch, Jon Bielby, Bárbara Martin-Beyer, Pedro Rincón, Francisco Correa-Araneda, Luz Boyero

**Affiliations:** 1 Research Unit of Biodiversity (CSIC, UO, PA), Oviedo University—Campus Mieres, Spain; 2 Centro de Investigación, Seguimiento y Evaluación, Parque Nacional de la Sierra de Guadarrama, Rascafría, Spain; 3 Museo Nacional de Ciencias Naturales-CSIC, Madrid, Spain; 4 Liverpool John Moores University, School of Natural Sciences and Psychology, Byrom Street, Liverpool, United Kingdom; 5 Unidad de Cambio Climático y Medio Ambiente, Instituto de Estudios del Hábitat (IEH), Facultad de Arquitectura y Construcción, Universidad Autónoma de Chile, Temuco, Chile; 6 Núcleo del Estudio en Ciencias Ambientales (NEA) y Departamento de Ciencias Ambientales (Facultad de Recursos Naturales), Universidad Católica de Temuco, Temuco, Chile; 7 Faculty of Science and Technology, University of the Basque Country (UPV/EHU), Vizcaya, Spain; 8 IKERBASQUE, Basque Foundation for Science, Bilbao, Spain; University of Idaho, UNITED STATES

## Abstract

Introduction of alien fish is a major problem for the conservation of amphibians inhabiting originally fishless mountain streams. While fish eradication programs in lakes and ponds have proven successful for the recovery of amphibian populations, there is no such information for stream-dwelling amphibians, possibly because fish removal from streams is difficult and costly. Here, we show the first case of successful recovery of a stream-dwelling amphibian (*Rana iberica*) in a mountain area of central Spain, following eradication of introduced brook trout (*Salvelinus fontinalis*) and native brown trout (*Salmo trutta*) translocated from downstream reaches by local anglers. Electrofishing for 12 consecutive years eradicated both fish species in the introduced area, and allowed the recovery of the *R*. *iberica* population as a result of natural recolonization from nearby streams and reintroduction of captive-reared individuals. Our results demonstrate how electrofishing can be a costly but effective method for the eradication of introduced fish and the conservation of stream-dwelling amphibians.

## Introduction

The introduction of alien species is one of the major causes of current extinction of amphibian populations and species [1]. Of special concern are fish, mainly trout species, which are often introduced for recreational fishing and can induce amphibian declines in protected areas [2, 3]. This is particularly important in mountain areas, which are originally devoid of fish due to the presence of physical barriers (e.g., cascades and steep stream reaches) that prevent natural fish colonization from downstream areas [4]. Trout are able to rapidly establish self-sustaining populations in introduced areas and spread to new locations downstream [5], negatively affecting amphibians due to predation, competition for resources, habitat degradation and/or disease transmission [6].

Mechanisms by which introduced fish can cause amphibian declines or extinctions are well known, but dealing with them is difficult [7], particularly when fish are introduced into streams. Gillnetting can be an effective method for fish eradication in lakes and ponds [8], allowing recovery of amphibian abundance and distribution to pre-introduction levels [3, 9, 10]. However, this method cannot be used in most streams, which are shallow and have high substrate complexity. Given that chemical methods such as rotenone are toxic for native organisms [8], other methods such as electrofishing, intensive angling or trojan Y fish, are more appropriate for removing introduced fish from streams.

However, the efficiency of electrofishing is variable, depending on the chemical and physical characteristics of streams (e.g., conductivity, temperature and habitat features) or the swimming behavior of fish species [11]. Multiple passes are usually necessary for fish removal [12], and eradication is often not achieved [13–15]. Thus, published reports of successful trout eradication from streams are rare [16–18] and, to our knowledge, there is no published evidence of recovery of amphibian populations due to electrofishing removals in streams.

Here, we report the first published case of recovery of a stream-dwelling native amphibian (the Iberian frog, *Rana iberica*) as a result of trout removal using electrofishing in streams of a mountain area in central Spain, the Peñalara Massif. *Rana iberica*, which is endemic from the Iberian Peninsula and endangered in mountain areas of central Spain [19], had been nearly extirpated from this area, due to the introduction of the North American brook trout (*Salvelinus fontinalis*) in the 1970s and the simultaneous translocation of the native brown trout (*Salmo trutta*) from downstream reaches by local anglers [20] (see Study area section). First, we investigate the efficiency of electrofishing through 12 consecutive years, until trout eradication was achieved. Second, we demonstrate the successful stream recolonization of *R*. *iberica* following fish eradication, due to natural recolonization and our reintroduction efforts in streams with and without nearby colonists, respectively. Our study provides crucial information for the management of stream-dwelling amphibian populations in mountain areas.

## Materials and methods

### Study area

**Fig 1:**
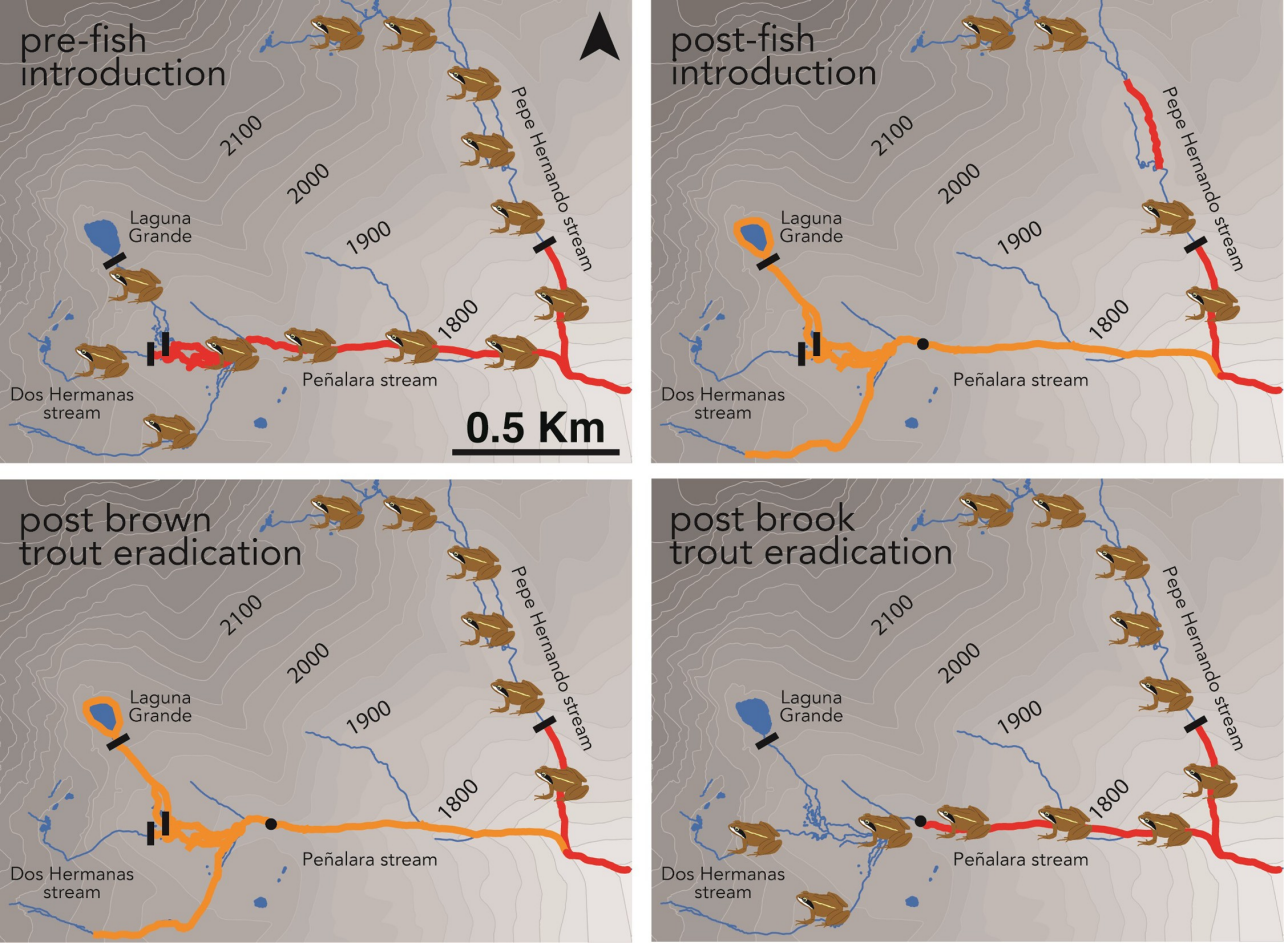
Progress of *Rana iberica* populations in the study area since pre–trout introduction times (before 1970’s) until present. Red and orange lines indicate distribution ranges of brown and brook trout, respectively. Black lines indicate probable natural upstream barriers for trout, and the black dot indicates the upper limit in Peñalara stream after a gauging station built in 1999.

The Peñalara Massif (Guadarrama National Park, central Spain, 40° 50’ N, 3° 57’ W) is an alpine area between 1,800 and 2,430 m asl containing more than 250 ponds and many small rivulets draining into two main streams, both less than 2 km in length: the Peñalara stream (PE) and the Pepe Hernando stream (PH). PE flows out of the Laguna Grande pond (surface area 5,452 m^2^, maximum depth 4.7 m), while PH flows through the adjacent small glacial valley (Fig 1).

Brook trout had been introduced in the Laguna Grande pond, from which it spread into PE and its tributary, Dos Hermanas (DH) stream (Fig 1), establishing a reproductive population with densities of 0.10 individuals/m^2^ [20]; it became rare downstream the confluence of PE and DH and was absent downstream the confluence of PE and PH, possibly due to inappropriate habitat conditions or to competition with brown trout [20]. Brown trout individuals had been translocated from their original downstream area into the higher part of PH, which was a naturally fishless reach because it was located upstream a steep section and a 1-m high vertical waterfall with no jump-pool at its bottom [20] (Fig 1). Brown trout established a small, isolated, but self-sustaining population in this area, with densities of 0.61 individuals/m^2^ [20].

The area originally supported large populations of native amphibians, including *R*. *iberica*. In many regions across the distribution of this species, adults and tadpoles coexist with brown trout in middle reaches of streams and small rivers, where gradual banks and shallow sections allow frogs to scape from trout; this includes areas surrounding the Peñalara Massif below 1600 m asl (i.e., outside the limits of Fig 1). In contrast, in the Peñalara Massif, *R*. *iberica* tadpoles and brown and brook trout prefer deeper habitats with slow water flow, avoiding riffles and cascades due to fast flow [20]. Frog tadpoles have been shown to respond to chemical cues from brown and brook trout by reducing their movement, although the response towards the native brown trout is stronger [20]. However, this antipredatory behaviour is probably unuseful in mountain pools with near vertical steep sides, where tadpoles are forced to share the habitat with trout permanently. Therefore, *R*. *iberica* had been nearly extirpated after fish introductions in the Peñalara Massif, only persisting in two smalls ponds next to PH that had not been invaded by trout (Fig 1) and in very few fishless ponds located in a higher, flat area of PH [20]. Finally, the few ponds occupied by the species upstream PH had experimented a sharp decline in the last two decades, possibly due to the chytrid fungus and/or climate change [19].

### Fish removal

Animal handling procedures were approved by the Consejería de Medio Ambiente, Comunidad Autónoma de Madrid, Spain (permit numbers: 10/059209.9/06, 10/025449.9/08, 10/168152.9/09,10/012157.9/10, 10/121009.9/11, 10/032921.9/12, 10/071126.9/13, 10/130923.9/14). Trout were removed from the Laguna Grande pond in 1998–2002 using gillnets, as part of another study [21]. In streams, we conducted yearly electrofishing campaigns for 12 consecutive years (2002–2013 in PH, 2003–2013 in PE and DH) in early September, when young-of-the year fish were large enough to be easily captured. A yearly campaign comprised usually less than 10 days in which we conducted 1–3 passes on every single inch of the studied sections using a DEKA 3000 “Lora” backpack electrofishing equipment, until no more individuals were captured. In PE we first covered an upstream section between a gauging station–which represented a physical barrier to fish migration–and the Laguna Grande pond (stream length sampled: 1,285 m), and then moved from the confluence of PE and PH up to the gauging station (stream length sampled: 1,882 m); in PH we started at the waterfall–which represented the natural downstream limit of the brown trout population–and moved upstream (stream length sampled: 815 m) (Fig 1).

### Amphibian surveys and reintroduction

Streams were surveyed for *R*. *iberica* individuals using visual encounters every 10–20 days, from June to late August, from 2002 to 2014. As there were no signs of natural recovery from 2002 to 2005 (i.e., the species was absent in the electrofished sections), we reintroduced the species in PE and DH (but not in PH, where it was present in nearby ponds that could allow natural recolonization). The reintroduction source was composed of tadpoles and clutches from a stream branch of PH that dried out every summer and prevented complete larval development. These tadpoles and clutches were taken to a rearing facility that was established in the surroundings of the National Park in 2008 [22]. Reintroduction was conducted in late August, from 2005 to 2014, by releasing captive-reared individuals at various metamorphosis stages, mostly froglets (newly metamorphosed individuals) but also tadpoles, juveniles (individuals that had not yet reached the size of sexual maturity) and adults. Additionally, we transferred tadpoles or clutches from the rearing facility to PE and DH. Numbers of individuals released and exact sites of release were decided every year based on observations. Although we first tried to mark individuals using elastomer tags, in order to be able to identify recaptured individuals, tags were inefficient due to the small size of individuals and we opted to stop marking them.

## Results

### Fish removal

**Fig 2:**
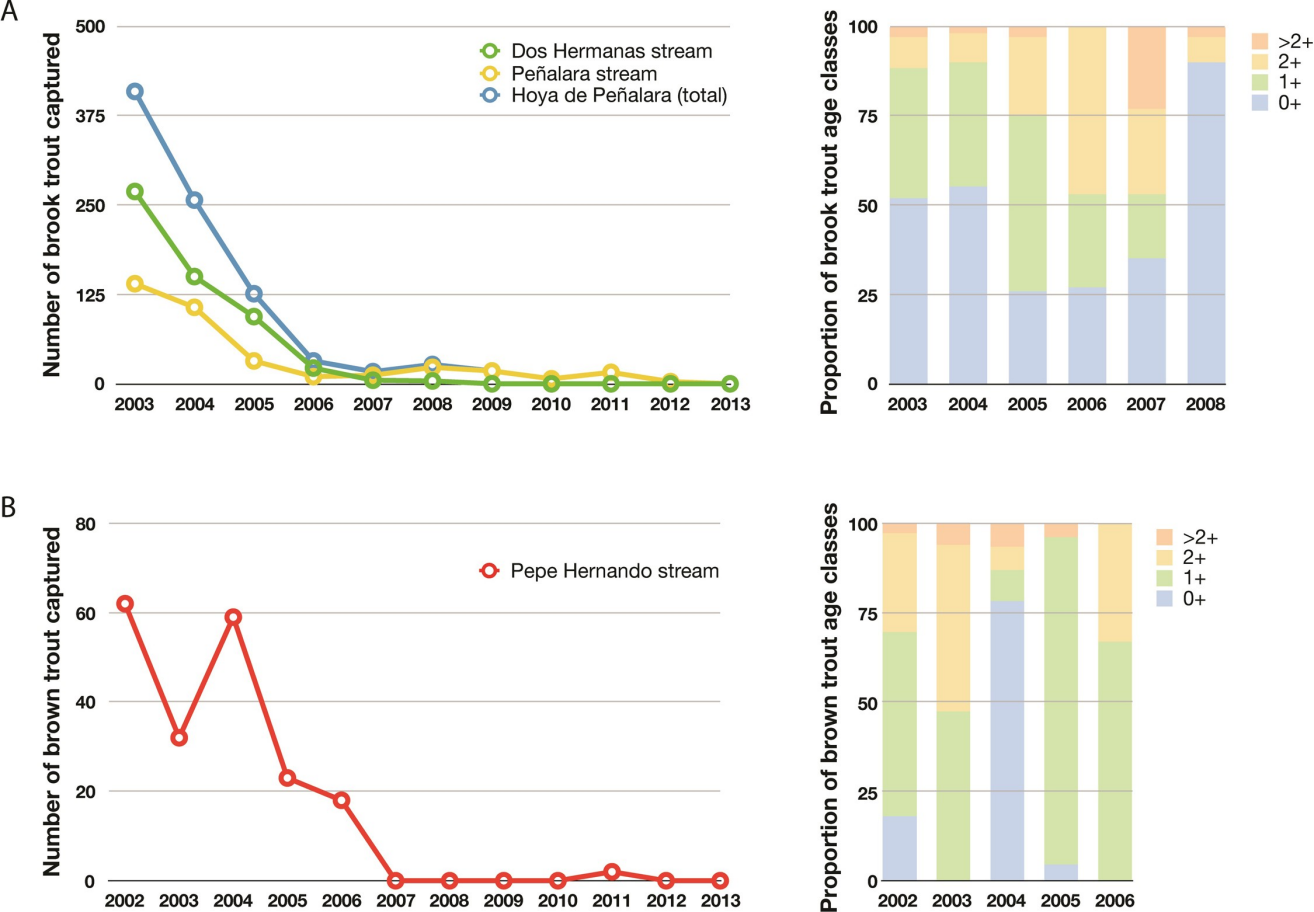
Number of individuals and proportion of age classes (0+, 1+, 2+ or >2+) of (A) brook trout and (B) brown trout captured in the study streams.

We captured 912 brook trout in PE and DH (368 and 544, respectively); most individuals were removed at the beginning of the campaign (e.g., 87% in the first 3 years), but eradication was achieved after 10 years (Fig 2A). In PH we captured 196 brown trout (78% in the first 3 years); in the next 2 years virtually all fish had been removed, with no captures in the following 7 campaigns, except in 2011 when 2 individuals were caught (Fig 2B). All brook trout captured were euthanised, while brown trout from PH were marked by adipose fin clipping [23] and released downstream their natural upstream limit. We did not remove brown trout from PE because their natural distribution limit upstream is unknown.

### Amphibian recolonization

**Fig 3:**
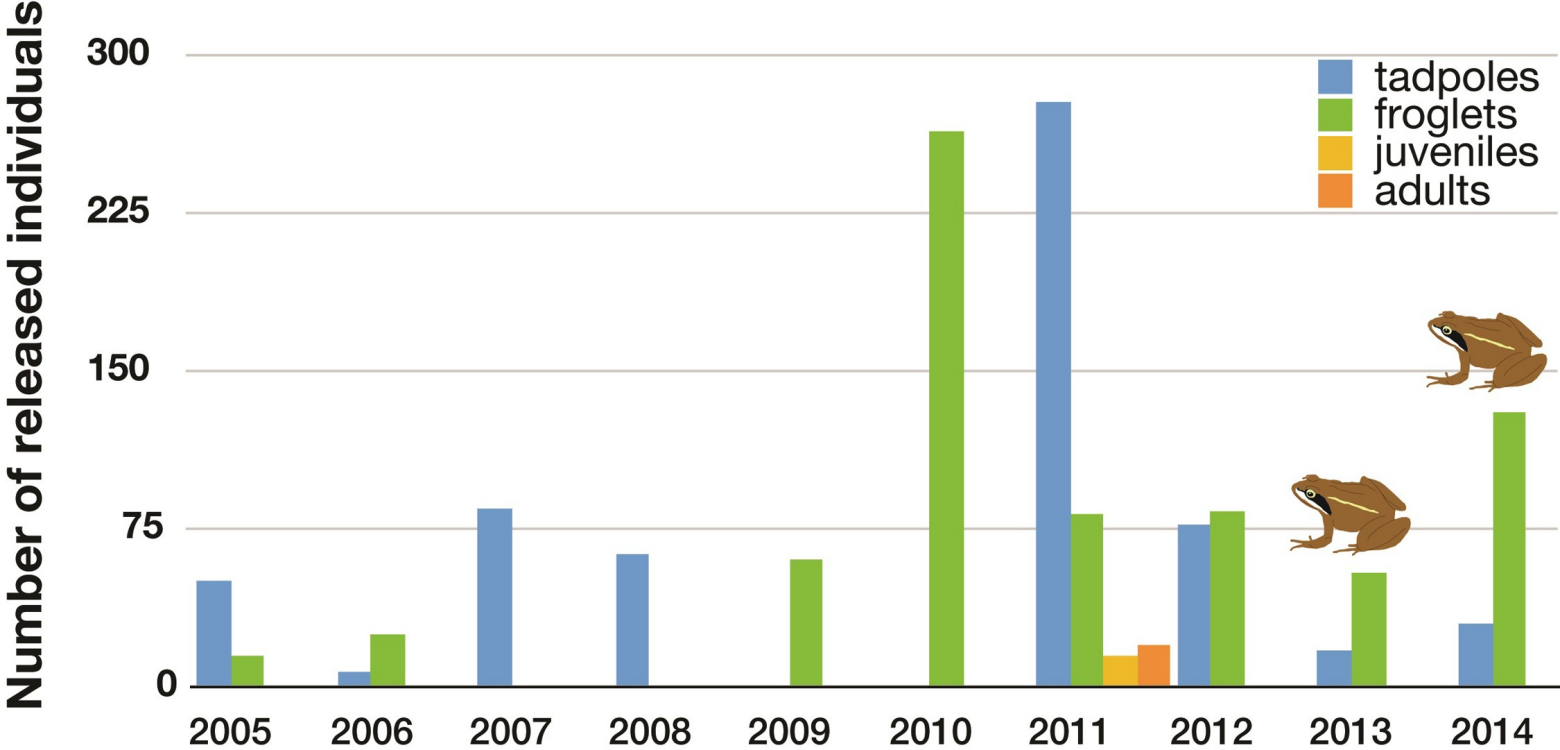
Number of individuals of *Rana iberica* released in Peñalara and Dos Hermanas streams. The frog icon indicates the years when adult frogs in breeding conditions or signs of reproduction were observed.

Froglets were released along PE and DH, while tadpoles–which are more vulnerable to predation–were released only in the upper section of DH until 2011 and, subsequently, when fish had almost been eradicated, along PE and DH. We released 32–395 individuals per year, and 1358 individuals in total (Fig 3).

Amphibian surveys showed that the electrofished section of PH was naturally recolonized in 2007, which was evidenced by the presence of tadpoles; froglets were found for the first time in 2008 in this area (Fig 1). Numbers of tadpoles and adults detected in successive years in the electrofished section of PH were similar to those of surrounding sections never invaded by trout (J.Bo., pers. obs.).

In PE and DH, we observed froglets and juveniles in 2005 and subsequent years; breeding adults and tadpoles were seen for the first time in 2013, which evidenced the occurrence of reproduction in the area (Fig 1). Although formal surveys ended in 2013, observations of breeding adults and tadpoles in subsequent years (2014–2018) suggest that *R*. *iberica* populations have established in both areas. The small number of individuals detected in PE and DH (no more than 50 tadpoles, 10 froglets and 2 adults in a single year) precluded formal analyses of frog recolonization, but were in accordance with the small densities exhibited by the species in the surrounding lower areas where it coexists with native brown trout (J.Bo., pers. obs.).

## Discussion

Our study shows that electrofishing can eradicate introduced trout in mountain streams. Few published studies have reported complete alien trout removal from streams using electrofishing [16–18], while this method has often been proven successful in lakes and ponds (e.g., [9, 8, 10]). Moreover, several studies have failed to achieve trout eradication from streams using electrofishing (e.g. [14, 15, 24]), and other removal methods are known to be ineffective due to stream physical characteristics (e.g., gillnetting) or because they are toxic for native fauna (e.g., chemical methods). Thus, here we provide one of the few successful reports of alien trout eradication exclusively from streams, which is of major importance for the conservation of stream-dwelling native populations.

Our trout removal program with yearly campaigns showed that most fish had been removed from the study stream reaches (with around 4 km electrofished in total) in the first 3 (brook trout) to 5 years (brown trout), with very low densities remaining until eradication on year 10 (brook trout) to 11 (brown trout). The other studies reporting successful electrofishing campaigns achieved trout eradication in 5 removal efforts (3 passes each) over 2 years for a 858-m long stream reach [16]; over 6 years for a 4.5-km stream reach, with 4–5 passes per year [18]; or over 4–8 years with multiple passes per year for several stream reaches ranging from 1.7 to 3.0 km long [17]. These results altogether indicate that electrofishing can be an effective method for removing invasive trout from streams over a period of several years, although the effort required for eradication is considerable.

A novel finding of our study is that, following trout eradication, stream-dwelling amphibian populations can successfully recover in areas where they had been dramatically reduced or disappeared. Several studies have reported similar situations in lakes–being the recovery of *Rana muscosa* in Sierra Nevada (USA) the most significant example [3, 9, 10]–but, to our knowledge, this is the first report for streams, with the only exception of lake inlet/outlet streams that were included in lake studies.

We showed that recolonization could occur naturally when nearby populations were present, as was the case in PH. *Rana iberica* had disappeared from the stream after the introduction of brown trout, given that both species were confined to pool habitats with lower water flow but also with vertical steep sides where amphibian larvae were not able to avoid predation [20]. Thus, before brown trout eradication, we never observed tadpoles in stream pools occupied by trout, or in lateral pools connected to an upstream area containing trout [20]. We saw *R*. *iberica* individuals in the electrofished area for the first time 5 years after trout removal, when trout densities had been drastically reduced. As we do not have data of *R*. *iberica* populations previous to fish introductions, we do not know the time required for population recovery, but data studies with *R*. *muscosa* in lakes have shown that frog populations may require >10 years to recover after fish disappearance [3].

In contrast to PH, the reintroduction of captive-bred individuals of *R*. *iberica* was necessary in PE and DH, where no nearby colonists were present. Our observation of froglets and juveniles from the first year suggest that recovery occurred within a very short time, although we cannot discard that individuals observed were those which had been released in the same year. In fact, we did not observe reproductive adults and tadpoles until year 9 of reintroduction.

The number of individuals released is an important variable for the success of amphibian reintroductions. Usually, success increases when large numbers of individuals (> 1,000) are released [25]. Our study supports this general rule, as we first detected *R*. *iberica* reproduction after 1,000 individuals had been released. Another critical, but little understood, issue influencing the success of amphibian reintroductions is the developmental stage of the released individuals [25]. The fact that we released mostly froglets and tadpoles allowed us to introduce larger numbers of individuals than if we had used juveniles or adults. Moreover, the introduction of adults often results in emigration, as they are philopatric to breeding sites, so introducing larval stages increases probability of success [26]. Still, the number of individuals found in PE and DH, where individuals were released, was low compared to those found in PH, where natural recolonization occurred, indicating that population recovery following reintroduction may take longer. However, the fact that we could not mark and recapture individuals precluded any formal analysis.

In summary, our study shows that stream-dwelling amphibians are able to recolonize the stream habitat formerly occupied, shortly after fish eradication, when nearby source colonists exist. In the absence of such colonists, reintroduction is required, and an adaptive management approach such as ours can be successful. Importantly, we monitored the *R*. *iberica* population during the entire eradication and reintroduction actions and afterwards–such long-term monitoring programs following reintroductions are uncommon, especially in mountain areas, but they can be critical for the success of reintroductions [27].
